# Production of Organic Acids by Probiotic Lactobacilli Can Be Used to Reduce Pathogen Load in Poultry

**DOI:** 10.1371/journal.pone.0043928

**Published:** 2012-09-04

**Authors:** Jason M. Neal-McKinney, Xiaonan Lu, Tri Duong, Charles L. Larson, Douglas R. Call, Devendra H. Shah, Michael E. Konkel

**Affiliations:** 1 School of Molecular Biosciences, College of Veterinary Medicine, Washington State University, Pullman, Washington, United States of America; 2 Department of Veterinary Microbiology and Pathology, College of Veterinary Medicine, Washington State University, Pullman, Washington, United States of America; Catalan Institute for Water Research (ICRA), Spain

## Abstract

Probiotic *Lactobacillus* can be used to reduce the colonization of pathogenic bacteria in food animals, and therefore reduce the risk of foodborne illness to consumers. As a model system, we examined the mechanism of protection conferred by *Lactobacillus* species to inhibit *C. jejuni* growth *in vitro* and reduce colonization in broiler chickens. Possible mechanisms for the reduction of pathogens by lactobacilli include: 1) stimulation of adaptive immunity; 2) alteration of the cecal microbiome; and, 3) production of inhibitory metabolites, such as organic acids. The *Lactobacillus* species produced lactic acid at concentrations sufficient to kill *C. jejuni in vitro*. We determined that lactic acid produced by *Lactobacillus* disrupted the membrane of *C. jejuni*, as judged by biophotonics. The spectral features obtained using Fourier-transform infrared (FT-IR) and Raman spectroscopy techniques were used to accurately predict bacterial viability and differentiate *C. jejuni* samples according to lactic acid treatment. FT-IR spectral features of *C. jejuni* and *Lactobacillus* grown in co-culture revealed that the metabolism was dominated by *Lactobacillus* prior to the killing of *C. jejuni*. Based on our results, the development of future competitive exclusion strategies should include the evaluation of organic acid production.

## Introduction

Probiotics are live microorganisms that confer a health benefit to a host [1], [2]. Perhaps the most commonly used probiotic species belong to the genus *Lactobacillus*. One potential probiotic benefit is improved resistance to enteric pathogens through competitive exclusion [3]. Commercial probiotic preparations containing lactobacilli are used in the egg and poultry industry to improve performance parameters, including mean egg weight, market-aged body weight, and feed conversion ratio [4], [5]. In this study, we sought to examine the probiotic activity of lactobacilli against *Campylobacter jejuni*.

The Gram-negative bacterium *Campylobacter jejuni* is a leading bacterial cause of food-borne illness. Each year, there are approximately 2.5 million cases of human campylobacteriosis in the United States and an estimated 400–500 million cases worldwide [6]. Infections are characterized by fever, abdominal cramps and diarrhea that may contain blood and leukocytes [6]. The ingestion of as few as 500 organisms may result in *C. jejuni* infection with symptoms appearing between 1–7 days after consumption of contaminated food or beverages [7]. While *C. jejuni*infections are typically self-limiting, they have been implicated as an antecedent for Guillain-Barré syndrome, an acute autoimmune-mediated polyneuropathy characterized by ascending paralysis [8].

*C. jejuni*is a common commensal organism of chickens, colonizing the cecum at up to 10^8^ CFU per gram of cecal contents [9], [10]. Contamination of chicken meat occurs during processing. One study detected *C. jejuni* on greater than 80% of chicken carcasses [11]. Consequently, a significant number of *Campylobacter* infections have been linked to the improper handling and consumption of poultry products [12], [13]. Strict biosecurity measures and improved hygienic practices at slaughter and have had limited success in reducing *C. jejuni* colonization of chicken flocks and contamination of chicken carcasses [14], [15]. One quantitative risk assessment indicates that the incidence of *C. jejuni* infection in humans could be reduced 30-fold if the number of *C. jejuni* in poultry was reduced 100-fold [16]. Thus, the effectiveness of experimental intervention measures such as oral administration of competitive exclusion organisms are being explored.

Competitive exclusion strategies for reducing *C. jejuni* colonization in chickens have been evaluated and resulted in varying levels of success. Oral administration of probiotic preparations containing *Klebsiella pneumoniae*, *Citrobacter diversus*, and *Escherichia coli* significantly reduce *C. jejuni* colonization of chickens [17], [18]. In addition, undefined cultures derived from cecal mucosa of chickens have been found to reduce colonization by *C. jejuni* more effectively than cultures derived from cecal contents [19], suggesting the ability of bacteria to adhere to the gastrointestinal mucosa is important for competitive exclusion organisms. Also, a commercial competitive exclusion product designed for use against *Salmonella* showed partial efficacy against *C. jejuni* [20]. Interestingly, strains of *C. jejuni* isolated from chickens can competitively exclude *C. jejuni* recovered from infected humans, when both are administered to chickens [21]. However, the safety of such a strategy is questionable given that all strains of *C. jejuni* are presumed to be pathogenic [22].

A probiotic preparation containing lactobacilli has been shown to reduce colonization and fecal shedding of *C. jejuni* in market-aged broiler chickens [23]. Researchers have shown that cell-free extracts derived from probiotics, including *L. acidophilus*, down-regulate gene expression from the *flaA* promoter of *C. jejuni*, suggesting that probiotic bacteria may influence the expression of genes important for chicken colonization [24]. The *flaA* gene encodes the major filament protein, and is required for motility [25]. While the administration of probiotic organisms appears to be a promising strategy to reduce *C. jejuni* colonization in poultry, further studies are needed to elucidate the mechanisms responsible for competitive exclusion of *C. jejuni* by *Lactobacillus* in chickens.

In this study, we evaluated the ability of four *Lactobacillus* strains (*L. acidophilus* NCFM, *Lactobacillus crispatus* JCM 5810, *Lactobacillus gallinarum* ATCC 33199 and *Lactobacillus helveticus* CNRZ32) to reduce colonization of *C. jejuni* in commercial broiler chickens. We also examined potential mechanisms responsible for competitive exclusion, including production of antagonistic metabolites (*i.e.,* lactic acid), modulation of antibody responses, and manipulation of the cecal microbiota. The most significant finding of this study is that *Lactobacillus* can dominate the metabolic activity of *C. jejuni* through the production of inhibitory organic acids, when the two organisms are grown in the same environment.

## Results

### Lactobacilli Inhibit *C. jejuni* Growth *in vitro*

**Figure 1:**
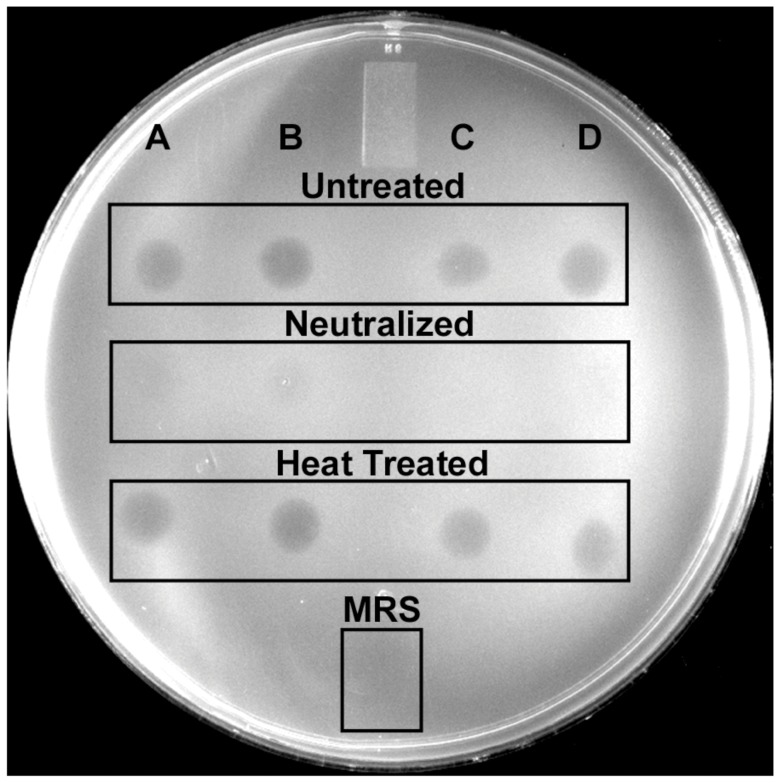
Inhibition of *C. jejuni* by lactobacilli. Overnight cultures of *C. jejuni* were inoculated into 10 ml MH soft agar, overlaid on MH agar, and incubated 24 h at 37°C. Supernatants from overnight cultures of *Lactobacillus* were left untreated, neutralized with 6.25 N NaOH, or heat treated (boiled). Treated supernatants were filter sterilized and spotted onto the *C. jejuni* inoculated soft agar. *Lactobacillus* strains used are indicated as follows: (A) *L. acidophilus*, (B) *L. crispatus*, (C) *L. gallinarum* and (D) *L. helveticus*.

The bacterial strains used in this study are listed in Table S1. The ability of four *Lactobacillus* species to inhibit the growth of *C. jejuni in vitro* was evaluated. Cultures of all four *Lactobacillus* strains limited the growth of *C. jejuni* when spotted onto lawns of six different *C. jejuni* strains, but the *C. jejuni* F38011 strain was found to be the most sensitive to the inhibitory effects (Table S2). In addition, *L. crispatus* most effectively inhibited the growth of all *C. jejuni* strains tested when compared with *L. acidophilus*, *L. gallinarum* and *L. helveticus* (*P = *0.008, Kruskal-Wallis Test). To determine if the production of organic acids, presumably lactic acid, contributes to the inhibitory ability of these lactobacilli, untreated and treated cell-free supernatants of *Lactobacillus* cultures were spotted on a lawn of *C. jejuni* (Figure 1). Neutralization of supernatants with NaOH greatly reduced inhibition of *C. jejuni* growth, suggesting that the inhibitory effect was due to acid production. Heat-treatment of supernatants did not affect the inhibition of *C. jejuni* growth, indicating that a heat-labile component was not responsible for the inhibition of *C. jejuni.* To address the possible contribution of bacteriocins in the observed inhibition of *C. jejuni* growth, plates spotted with *Lactobacillus* cultures were treated with either trypsin or proteinase K prior to being overlaid with top agar containing *C. jejuni*. Protease treatment of the supernatants did not reduce inhibition of *C. jejuni* (not shown), suggesting that inhibition was not due to the production of a proteinaceous component. These data support the hypothesis that the ability of *Lactobacillus* cultures to inhibit growth of *C. jejuni in vitro* is due, at least in part, to the production of lactic acid.

### Oral Administration of *Lactobacillus* Reduced *C. jejuni* Colonization of Broiler Chicks

**Figure 2:**
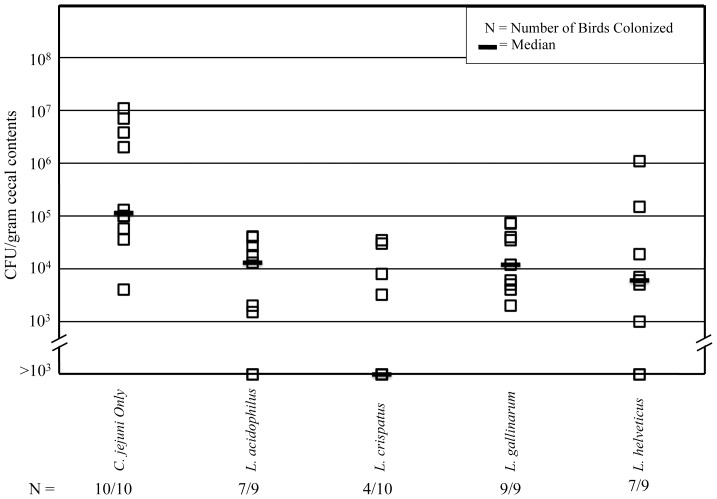
*Lactobacillus* reduces *C. jejuni* colonization of broiler chicks. Broiler chicks were administered *Lactobacillus* by oral gavage (∼10^8^ CFU) at day of hatch and 4 days post-hatch. Chicks receiving *C. jejuni* challenge were administered *C. jejuni* F38011 by oral gavage (∼10^8^ CFU) at day 14 post-hatch. Chickens were euthanized and necropsied at seven days post-challenge. Cecal contents were serially diluted and plated onto Campy Cefex agar for enumeration of *C. jejuni*. No *C. jejuni* were detected in non-challenged chickens.

Commercial broiler chicks were administered lactobacilli as potential competitive exclusion organisms on day-of-hatch and four days post-hatch, and challenged with the *C. jejuni* F38011 strain at 14 days post-hatch. The chickens were euthanized and necropsied at 21 days post-hatch (7 days post-challenge with *C. jejuni*). *C. jejuni* present in the cecum of each chicken were enumerated (Figure 2). *C. jejuni* was not detected in the ceca of unchallenged chickens, indicating containment procedures were effective. All birds in the *C. jejuni* positive control group were colonized (*i.e., C. jejuni* only group). In the groups receiving *L. acidophilus* and *L. helveticus*, *C. jejuni* was detected in 7/9 chickens. Chickens administered *L. gallinarum* resulted in 9/9 birds colonized with *C. jejuni*, respectively. Overall, chickens receiving *L. crispatus* were colonized the least by *C. jejuni,* with only 4/10 birds colonized. Although treatment with all four strains of *Lactobacillus* bacteria reduced the median level of colonization with *C. jejuni* compared to uninoculated birds, *L. crispatus* was the most effective in reducing the number of chickens colonized with *C. jejuni*. The experiment was repeated using five birds per treatment group, and the trend in colonization was reproducible (not shown).

### Inoculation with *Lactobacillus* does not Significantly Perturb the Dominant Cecal Microbiota

To determine whether inoculation of chickens with *Lactobacillus* altered the cecal microbiota, cecal specimens from several treatment groups were selected for microbiome analysis, using16S rDNA sequencing (Table S3). The 16S rDNA was extracted from the cecal contents and sequenced, then classified using the RDP Classifier (Table S4). Of the 747 16S rDNA clones examined, 644 (86%) were classified as belonging to the phylum Firmicutes, 94 (13%) were classified as Bacteroidetes, 8 (1%) were unclassified, and a single clone was classified as a Proteobacteria. The Firmicutes were the dominant phylum observed, with the Clostridia being the major class across all the specimens accounting for 64% of the total clones in the libraries. While clones classified as *Lactobacillus* were identified in samples from groups receiving *L. crispatus*, *L. gallinarum*, and *L. helveticus*, *Lactobacillus* sequences were identified most often in the *L. crispatus* samples. The single Proteobacteria clone belonged to the genus *Salmonella,* which was found in the sample receiving *L. gallinarum* and challenged by *C. jejuni*. No clones were classified that belonged to the genus *Campylobacter*. Gram-positive flora were dominant across all cecal samples regardless of treatment with *Lactobacillus*. The major flora, aside from the increased number of *Lactobacillus* clones identified, predominated regardless of treatment.

### Anti-*C. jejuni* Antibody Titers were not Affected by Inoculation with *Lactobacillus*

Levels of anti-*C. jejuni* antibodies in sera at one week post-challenge were determined by ELISA (Figure S1). Microtiter plates were coated with 0.2 µg/well of *C. jejuni* F38011 whole-cell lysate, and the reactivity of chick sera was determined. Sera from the uninoculated chickens were used to determine the baseline level of nonspecific antibody reactivity. At 21 days post-hatch (7 days post-challenge) there was no difference in the reactivity of sera from the *C. jejuni*-inoculated groups compared to *C. jejuni* uninoculated (*i.e.,* naïve) chickens or chickens that received *C. jejuni* only. The presence or absence of the *Lactobacillus* strains did not correlate to a differential antibody response. Antibodies reactive to *Clostridium perfringens* alpha-toxin present in the chicken sera were also examined, to determine whether the *Lactobacillus* stimulated the production of natural antibodies (Figure S2). Antibodies against alpha-toxin were not detected in significant quantities and did not appear to be affected by the *Lactobacillus* strain administered. Collectively, these data indicate that stimulation of adaptive immunity was not responsible for the reduction of *C. jejuni* in chicks inoculated with *Lactobacillus.*

### Lactic Acid Produced by *Lactobacillus* Inhibits and Kills *C. jejuni*

To determine whether lactic acid in culture supernatants could be responsible for the *in vitro* growth inhibition of *C. jejuni* by *Lactobacillus*, we first determined the amount of D- and L-lactic acid produced by each of the four *Lactobacillus* strains. After 48 h of anaerobic growth (no aeration) the concentrations of D- and L-lactic acid were measured using a colorimetric enzymatic assay (Table 1). The supernatants of each strain contained nearly equivalent concentrations of the D- and L-lactic acid isomers. The supernatants of *L. crispatus* contained the most lactic acid (188±1.1 mM D-lactate and 158±10.6 mM L-lactate) while the supernatants of *L. acidophilus* contained the least lactic acid (130±4.9 mM D-lactate and 148±2.3 mM L-lactate).

**Table 1:** Production of lactic acid^a^.

Species	D-Lactate (mM)	L-Lactate (mM)
*L. acidophilus*	130±4.9^a^	148±2.3
*L. crispatus*	188±1.1	158±10.6
*L. gallinarum*	167±3.3	152±9.1
*L. helveticus*	177±3.8	182±4.9

a ±represent one standard deviation from the mean of triplicate assays.

**Figure 3:**
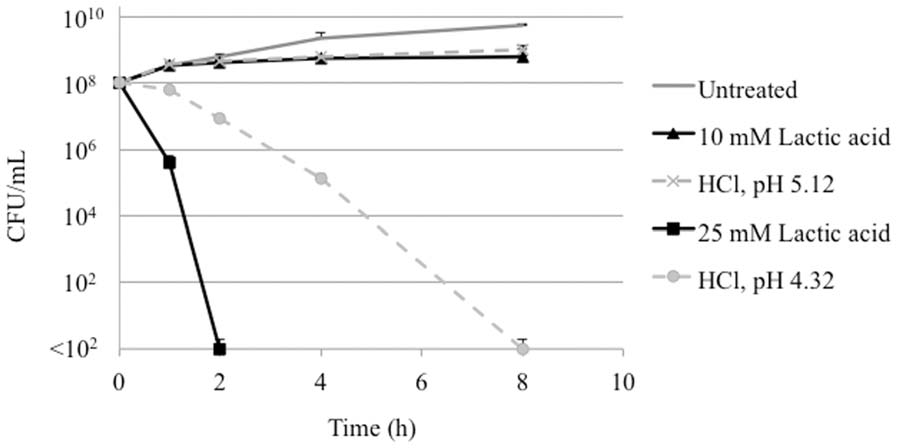
Lactic acid kills *C. jejuni in vitro*. An overnight MH broth culture of *C. jejuni* was inoculated into fresh media to an O.D._600_ of 0.1. Each broth culture was either untreated, supplemented with 10 mM, 25 mM, or 100 mM lactic acid, or supplemented with hydrochloric acid to achieve a pH equivalent to the lactic acid treated cultures (pH 5.12, 4.32, and 3.46, respectiveley). Samples were serially diluted and plated onto MHB agar for enumeration at 1, 2, 4, and 8 h after incubation. No viable *C. jejuni* were detected after 1 h when the media was supplemented with 100 mM lactic acid or acidified to pH 3.46 using HCl, therefore these data are not displayed.

To determine whether the inhibition of *C. jejuni* by lactic acid was solely pH-dependent, a survival experiment was performed using *C. jejuni* grown in MH broth that was either untreated, or supplemented with 10, 25, or 100 mM lactic acid. MH broth was also treated with HCl to reach an equivalent pH to media containing 10, 25, or 100 mM lactic acid (pH 5.12, 4.32, and 3.46, respectively). Five ml of each broth were inoculated with *C. jejuni* in triplicate, and samples were taken at 1, 2, 4, and 8 h for bacterial enumeration (Figure 3). Media containing 100 mM lactic acid or pH 3.46 (HCl) killed all *C. jejuni* within one h (not shown). Media containing 25 mM lactic acid resulted in the death of all *C. jejuni* by 2 h, while treatment media at pH 4.32 (HCl) did not kill all *C. jejuni* until 8 h. Media containing 10 mM lactic acid or pH 5.12 (HCl) inhibited *C. jejuni* growth slightly compared to the untreated medium. Collectively, the data show that lactic acid inhibits and kills *C. jejuni* through pH-dependent and pH-independent mechanisms.

### Lactic Acid Targets the Cell Membrane of *C. jejuni*

**Figure 4:**
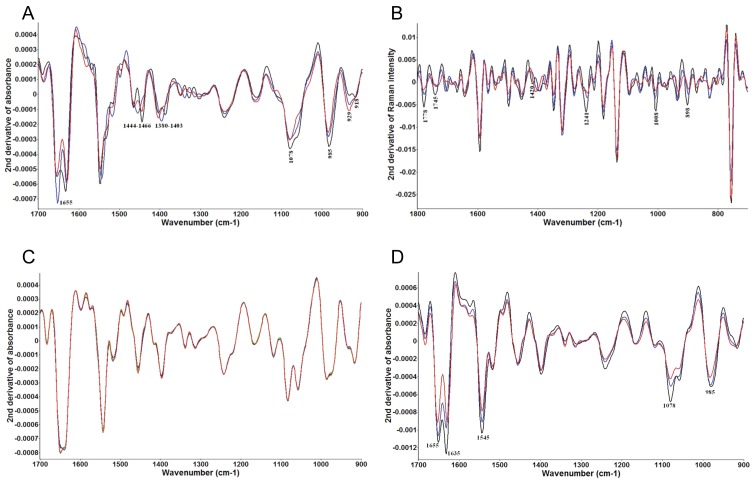
Lactic acid treatment of *C. jejuni* alters membrane structures. We performed FT-IR and Raman spectroscopy on *C. jejuni* and *L. crispatus* incubated for 1 h in broth containing various concentrations of lactic or hydrochloric acid. The second derivative transformations of the spectra are shown in Panels A–D. Panels A and B show the FT-IR and Raman spectra, respectively, of *C. jejuni,* while Panel C shows the FT-IR spectra of *L. crispatus.* In each experiment, the bacteria were incubated in broth containing 0 mM (black), 25 mM (blue), or 100 mM (red) lactic acid. Panel D contains the FT-IR spectra of *C. jejuni* grown in media that was untreated (pH 7.3), or treated with HCl to achieve a pH of 4.32 (black) or 3.46 (red). Wavenumbers are adjacent to spectral features of interest, and the chemical structures represented by each are summarized in the text and listed in Table S5.

Samples of *C. jejuni* grown for 1 h in media containing lactic acid were examined using complementary FT-IR and Raman spectroscopies to determine the mode of bacterial inactivation (Figure 4A and 4B, respectively). The detailed band assignments of significant (*P*<0.05) spectral variations are summarized in Table S5. Significant spectral variations were observed in chemical structures associated with DNA, mono- and polysaccharides, structural proteins, and fatty acids derived from membrane phospholipids. The FT-IR spectra of *L. crispatus* treated with lactic acid was used as a negative control, and no significant (*P*>0.05) spectral variations were observed (Figure 4C). These data indicate that lactic acid only affects *C. jejuni* and not *Lactobacillus*. Figure 4D shows the FT-IR spectral variations of *C. jejuni* treated with hydrochloric acid. In contrast to treatment with lactic acid, the spectra did not reveal alterations to the phospholipid components. These data indicate that lactic acid destabilizes the membrane of *C. jejuni,* and this antimicrobial effect is not solely pH-dependent. Raman spectroscopic based cluster analysis and dendrogram models were able to segregate *C. jejuni* samples according to treatment with selected concentrations of lactic acid, demonstrating the discriminatory ability of this technique (Figure S3). As the lactic acid detected in supernatants was produced by pure cultures over 48 h, we sought to determine the effects of *Lactobacillus* on the growth of *C. jejuni* when the organisms were grown together (prior to accumulation of lactic acid).

### *L. crispatus* Inhibits the Growth of *C. jejuni* During Co-culture

**Figure 5:**
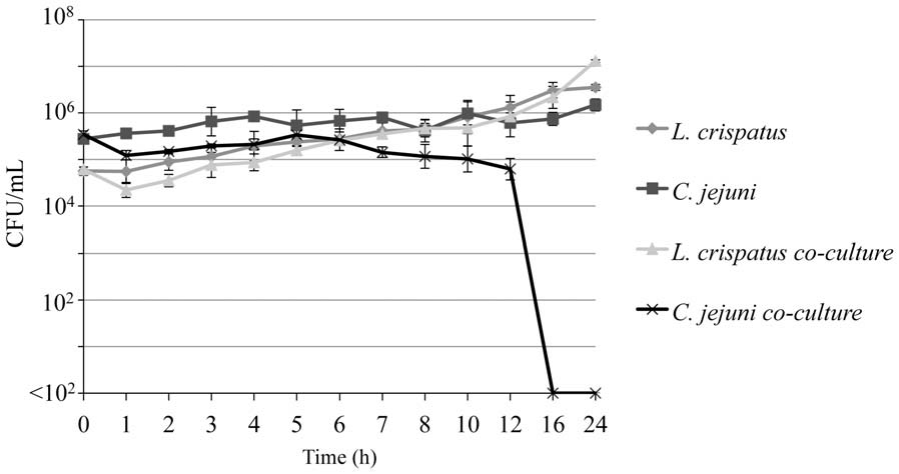
*L. crispatus* kills *C.* jejuni in co-culture. *C. jejuni* and *L. crispatus* were inoculated into pasteurized milk samples either in mono-culture (pure) or in co-culture and incubated for 24 h. The average number of CFU ± standard deviation is shown for each time-point.

We established a model to examine the metabolic activity of *C. jejuni* and *L. crispatus* in co-culture, using pasteurized milk as a model growth medium. This *in vitro* system is ideal as both organisms are metabolically active in this medium. *C. jejuni* and *L. crispatus* were inoculated into pasteurized milk either in monoculture or in co-culture and grown for up to 24 h. A survival curve of the pure and co-cultures is shown in Figure 5. While *L. crispatus* was able to grow at a rate in co-culture similar to that in pure culture, the growth of *C. jejuni* was significantly inhibited by *L. crispatus,* and no viable *C. jejuni* were detected at 16 h.

Visual inspection of the spectra obtained using FT-IR spectroscopy for the two bacterial species in monoculture and/or in co-culture revealed only minor differences between spectra (Figure S4). Prediction of viable *C. jejuni* in pure and co-culture based on FT-IR spectra was performed using a partial least squares regression (PLSR) model as described in previous work [26] (Figure S5). Table S6 shows the mean numbers (CFU/ml) and model parameters for *C. jejuni* in the different growth environments analyzed by PLSR. A good PLSR model should have a high value of R (>0.9) and RPD (>5) and low value for RMSE (<1). Overall, the PLSR model of *C. jejuni* viability in pure and co-culture provided a similar predictive capacity. Collectively, these data demonstrate that *Lactobacillus* inhibits *C. jejuni* growth *in vitro*, and the viability of *C. jejuni* in pure and co-culture can be accurately predicted by FT-IR spectral features.

### *L. crispatus* Alters the Metabolism of *C. jejuni* in Co-culture

**Figure 6:**
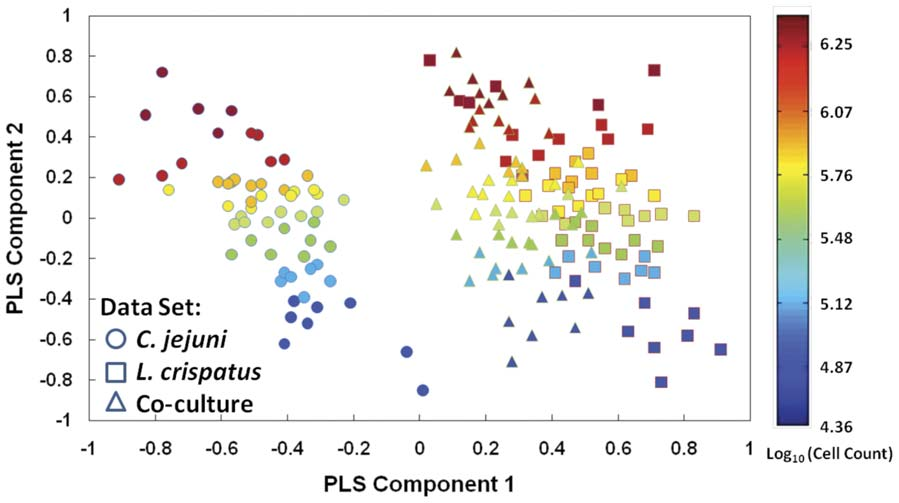
*L. crispatus* dominates the metabolic activity in co-culture. Partial least squares 2-component (PSL2) models were calibrated with pure monocultures of *C. jejuni* (circles) and *L. crispatus* (squares), and co-culture samples (triangles) were projected into the PLS2 model. Color spectrum represents log_10_ CFU/mL (cell count).

To determine whether the metabolic spectral fingerprints from the co-cultured samples were more similar to those of the pure cultures, we established a hybrid PCA-PLSR model to study bacterial metabolic activity in bacterial co-culture system [27]. Based upon the first two principal components extracted from multivariate analytical models, we conducted a PCA-PLSR projection to study bacterial metabolic activity in co-culture (Figure 6). The co-culture spectral cluster was adjacent to and partially overlapping with the spectral cluster for *L. crispatus* monoculture. This indicates that the metabolic activity in the co-culture was dominated by *L. crispatus*. Further, the first principal component (accounting for ∼70% of segregation) corresponded to spectral bands around 1100 cm^−1^, which are assigned to carbohydrates [26] and would be associated with *L. crispatus* metabolic activity and production of lactic acid from the utilized carbohydrate. Thus, the production of lactic acid is likely responsible for the dominant metabolic activity of *L. crispatus* compared to *C. jejuni* in co-culture.

#### Sub-lethal concentrations of lactic acid inhibit *C. jejuni* growth

In Figure 3, we demonstrated that lactic acid could kill stationary phase cultures of *C. jejuni*. To confirm that the inhibition of *C. jejuni* metabolism in co-culture with *L. crispatus* was due in part to lactic acid, we treated mid-logarithmic phase cultures of *C. jejuni* with sub-lethal concentrations of lactic acid (Figure S6). After 8 h of growth, samples treated with 5 or 10 mM lactic acid had ∼10 fold less *C. jejuni* than untreated samples. *C. jejuni* treated with 20 mM lactic acid died rapidly, and were undetectable at 4 h. This experiment demonstrates that lactic acid can inhibit the metabolism of *C. jejuni* at concentrations that are sub-lethal.

## Discussion

The colonization of livestock animals by pathogenic bacteria is a major source of human foodborne illness. While several intervention strategies have been implemented to reduce pathogen loads in livestock (*e.g.,* vaccination, antibiotic treatment, chemical disinfection, etc.), the oral administration of probiotic bacteria is advantageous, as they are easy to administer, inexpensive to produce, and may persist in the animal. We evaluated the ability of putative probiotic lactobacilli to inhibit the growth of *C. jejuni in vitro* and to competitively exclude *C. jejuni* colonization of commercial broiler chickens *in vivo*. All four strains of *Lactobacillus* tested reduced the median level of colonization by *C. jejuni* compared to the untreated control group, with *L. crispatus* treatment resulting in only 4/10 chickens colonized. Our results showed the number of *C. jejuni* was reduced by almost two orders of magnitude. We investigated possible mechanisms for the reduction of *C. jejuni* by *L. crispatus,* including: 1) production of bacteriocins; 2) stimulation of antibody production; 3) alteration of the cecal microbiome; and 4) production of lactic acid.

Of the possible mechanisms investigated, only the production of lactic acid was supported by our data. While the genomic sequences of *L. crispatus* JCM 5810, *L. gallinarum* ATCC33199, and *L. helveticus* CNRZ32 are unknown, *L. acidophilus* NCFM has been shown to produce the bacteriocin lactacin B [28]. This bacteriocin does not have activity against Gram-negative organisms, such as *C. jejuni*. Although a few strains of *Lactobacillus* are known to produce bacteriocins that are inhibitory to *C. jejuni* [29], [30], a proteinaceous component did not appear to be responsible for the anti-*C. jejuni* effects observed in this study. Noteworthy is that a similiar assay was used by others to screen >1200 *Lactobacillus* strains, only one of which was further characterized as producing an anti-*C. jejuni* bacteriocin [30]. Additionally, while probiotics have been reported to have immunostimulatory [31], [32] and adjuvant-like [33], [34] effects in chickens, administration of probiotic lactobacilli did not affect antibody responses to *C. jejuni*. Molecular analysis of the cecal microbiome of chickens found administration of lactobacilli did not dramatically perturb the major flora. Due to the limited number of 16s rDNA clones sequenced, it is possible that the lactobacilli may have altered less dominant microflora that were not able to detect. All of the probiotic strains examined in this study produced high levels of lactic acid that inhibited *C. jejuni* growth *in vitro.* Using complementary vibrational spectroscopies, we found that lactic acid targeted the membrane of *C. jejuni*, and that the inhibitory effect was not solely pH-dependent. Experiments performed with *C. jejuni* and *L. crispatus* in co-culture revealed that *L. crispatus* dominated the metabolic processes of the culture, leading to inactivation and death of *C. jejuni.* The results obtained support the hypothesis that the production of lactic acid by *Lactobacillus* is a likely mechanism for the reduction of *C. jejuni in vivo*.

The ability of *Lactobacillus* spp. to inhibit the growth of *C. jejuni in vitro* has been demonstrated previously [30], [35], [36], [37]. Few studies, however, have compared multiple lactobacilli and the elucidation of the mechanisms responsible for inhibition. In previous work, others found that hydrogen peroxide, acetic acid and lactic acid reduced *C. jejuni* counts in suspension and on chicken meat [38]. Neutralizing the pH of the *Lactobacillus* culture supernatants nearly abolished their inhibition of *C. jejuni,* indicating that the inhibition was mediated by lactic acid. The four strains of *Lactobacillus* examined in this study are all classified as obligate homofermenters, because they ferment glucose to lactic acid without the production of ethanol or additional organic acids [39]. Each strain produced greater than 250 mM lactic acid after 48 h fermentative growth in MRS broth. Survival experiments performed with *C. jejuni* revealed that 10 mM lactic acid significantly reduced the growth of *C. jejuni*, while 25 mM lactic acid resulted in the death of all *C. jejuni* by 2 h. The bacteriocidal effect was not completely pH-dependent, as media adjusted to the same pH with HCl had significantly less bacteriocidal activity. These results indicate that lactic acid is the predominant mechanism of *C. jejuni* inhibition *in vitro*.

The mechanism of inactivation of *C. jejuni* by lactic acid secreted from *L. crispatus* was investigated using complementary vibrational spectroscopies. FT-IR and Raman spectroscopies revealed that *C. jejuni* treated with lactic acid displayed alterations in spectral features corresponding to structural phospholipids found in the cell membrane. The drastic effect on the phospholipids was not observed in samples of *C. jejuni* treated with HCl. Additionally, the spectral features of *L. crispatus* were not affected by lactic acid, as was expected. These data strongly validated that the unique antimicrobial mechanism of lactic acid was not simply pH-dependent. The disruption of *C. jejuni* membrane integrity is in agreement with previous studies that used lactic acid to inactivate *Escherichia coli, Salmonella enterica*, and *Pseudomonas aeruginosa* [40].

Of the strains examined, *L. crispatus* appears to have the greatest probiotic activity. Inoculation of chicks with *L. crispatus* resulted in the greatest reduction in colonization, as only 4/10 birds were colonized, and none were colonized at levels ≥10^5 ^CFU/gram. Additionally, *L. crispatus* was the most effective at inhibiting the *C. jejuni* F38011, 81–176, RM1221, and Turkey strains *in vitro* (Table S2). *L. crispatus* also produced more acid than *L. acidophilus* and *L. gallinarum.* Only inoculation with *L. crispatus* increased the number of lactobacilli detected by 16s rDNA sequencing, indicating that it may be able to establish persistent colonization and become part of the dominant microflora. As *L. crispatus* appeared to be the most effective strain tested, we chose to examine the interaction between *C. jejuni* and *L. crispatus* when grown in the same environment.

FT-IR spectroscopy was also used to monitor the chemical composition of *L. crispatus* and *C. jejuni* co-cultures. Milk was chosen as the growth medium to examine co-cultures of *C. jejuni* and *L. crispatus*, as it is an *in vitro* system in which both bacteria are metabolically active. The two bacterial species were investigated both in monoculture and in co-culture in pasteurized milk during the first 24 h of growth. *C. jejuni* growth was inhibited after 6 h in co-culture, and was eliminated after 16 h. Metabolic fingerprinting of the co-culture by FT-IR was performed using samples taken after 6 h, as the number of viable *C. jejuni* and *L. crispatus* were generally equivalent at this time point. The metabolic profile of this dual bacterial culture was closer to that of pure *L. crispatus* cultures, indicating that *L. crispatus* dominated the metabolic activity in co-culture. Because *L. crispatus* and *C. jejuni* are not predicted to utilize the same nutrients in this medium, the inhibition of *C. jejuni* metabolism was presumably due to the production of inhibitory metabolites. Given that *C. jejuni* growth is inhibited by 5 mM lactic acid and killed by 20 mM, we propose that small concentrations of *L. crispatus* metabolites inhibit *C. jejuni* in co-culture, and the eventual build-up of lactic acid kills *C. jejuni*. Similar inhibition of *C. jejuni* metabolism may occur in the complex environment of the chicken gastrointestinal tract.

In summary, we have demonstrated that *L. crispatus* JCM 5810 is an effective competitive exclusion organism for *C. jejuni,* as evidenced in a reduction in the total number of *C. jejuni* colonized chickens and lower microbial load. The data from our *in vitro* experiments indicate that the production of lactic acid by *L. crispatus* is a likely mechanism for the reduction of *C. jejuni* colonization in chickens. The results of this study indicate that the production of antagonistic metabolites, such as lactic acid, is an important factor that must be considered in designing competitive exclusion strategies to reduce pathogen loads in livestock. Based on our findings, we hypothesize that *Lactobacillus* could be used to reduce the number of other microbes; any pathogen that is sensitive to lactic acid could be inhibited by *Lactobacillus* as well. Pathogens cannot use phase variation to escape the effects of lactic acid, and the development of lactic acid resistance would require multiple evolutionary steps. A future direction of this research will focus on identifying the changes in chemical composition and minor microbial populations within the cecum in response to inoculation with probiotic *Lactobacillus.*

## Materials and Methods

### Bacterial Strains and Growth Conditions

The bacterial strains used in this study are listed in Table S1. *Lactobacillus* strains were propagated statically at 37°C in deMan, Rogosa and Sharpe (MRS) [41] broth (Difco) or on MRS agar plates under microaerobic conditions (85% N_2_, 10% CO_2_, 5% O_2_). *C. jejuni* strains were cultured under microaerobic (85% N_2_, 10% CO_2_, 5% O_2_ ) conditions in Mueller-Hinton (MH) (Difco Inc., Detroit, MI) broth or on MH agar plates supplemented with 5% citrated bovine blood (MHB agar plates) at 37°C. Cultures were subcultured to a fresh plate every 24 to 48 h. Motility of *C. jejuni* cultures was confirmed prior to inoculation in chickens. *Escherichia coli* were grown on Luria-Bertani (LB) media (Difco) supplemented with ampicillin (100 µg/ml) when appropriate.

### Growth Curve Analysis

Overnight cultures of *Lactobacillus* strains were used to inoculate MRS broth. Growth was monitored at an absorbance of 600 nm (A_600_) using a BioscreenC analyzer (Growth Curves USA, Inc., Piscataway, NJ). Maximum growth rate (µ_m_) was determined by fitting the growth curves to a modified Gompertz [42] model using Prism 5.0 (Graphpad Software, Inc., La Jolla, CA).

### *C. jejuni* Inhibition Assays

Inhibition of *C. jejuni* cultures by lactobacilli was evaluated using spotted cultures and supernatants (cell-free media). For spotted cultures, overnight cultures of lactobacilli were spotted onto Brain Heart Infusion Agar (Difco) supplemented with 0.1% Tween 80 (Fisher Scientific, Hampton, NH) (BHI-T) and incubated overnight under microaerobic conditions. Subsequently, plates were overlaid with 10 ml molten MH soft agar (0.75%) inoculated with 100 µl of *C. jejuni* cultures standardized to A_540_ of 1.0 in MH broth. Plates were incubated for 24 h at 37°C under microaerobic conditions. Growth inhibition was evaluated by measuring the zones of inhibition around the *Lactobacillus* cultures and expressed as the ratio of the zone of inhibition to the zone of *Lactobacillus* colony growth in mm. To determine if bacteriocins contributed to inhibition, plates were treated with proteinase K (20 µg/µl) (Invitrogen, Carlsbad, CA) or trypsin (Sigma-Aldrich, St. Louis, MO) (10 µg/µl) prior to being overlaid with *C. jejuni*. To determine if peroxides contributed to inhibition, plates were treated with catalase (10 µg/µl) (Sigma) prior to being overlaid. Cells were pelleted from *Lactobacillus* cultures by centrifugation, and the supernatants were collected and either boiled for 6 min, neutralized to pH 7 with 6 N NaOH (Fisher), or left untreated. Supernatants were subsequently filter sterilized (0.22 µm) and spotted onto solidified MH soft agar inoculated at 1% with *C. jejuni*, and incubated overnight.

### Chicken Colonization Experiments

Chicken colonization studies were performed as described previously [43]. Briefly, *Lactobacillus* cultures were grown statically in MRS at 37°C for 18 h. The *C. jejuni* F38011 strain was cultured in MH broth at 37°C for 18 h prior to inoculation. Ten groups of chicks were used, each containing ten individuals. Group 1 was kept as the uninoculated control group (*i.e.,* negative control for *C. jejuni* colonization). On the day of hatch and four days post-hatch, the remaining nine groups of chicks were inoculated by oral gavage with 0.5 ml lactobacilli suspension (∼10^8^) as follows: group 2 and 6, *L. acidophilus* NCFM; group 3 and 7, *L. crispatus* JCM5810; group 4 and 8, *L. gallinarum* ATCC 33199; groups 5 and 9, *L. helveticus* CNRZ32. Group 10 was inoculated with PBS. At 14 days post hatching, Groups 5–10 were administered *C. jejuni* F38011 by oral gavage with 0.5 ml bacterial suspension (∼10^8^ CFU). The chickens in each group were euthanized and necropsied at day 21 (one week post-challenge). One cecum and the intestine were dissected from each chicken. The samples were weighed, diluted in an equal volume (w/v) of MH, and thoroughly stomached. Samples were serially diluted in MRS and MH broth for enumeration of *Lactobacillus* and *C. jejuni*, respectively. The MRS dilutions were plated onto Rogosa SL (Difco) agar plates for enumeration of lactobacilli while MH dilutions were plated on Campy Cefex (Difco) agar plates for enumeration. The chicken colonization experiment was repeated using five birds per group to ensure reproducibility. The chicken experiments were performed at the Washington State University Avian Health and Food Safety Laboratory in Puyallup, WA, and at the poultry isolation facility in Pullman, WA. All animal experiments were performed using protocols approved by the Institutional Animal Care and Use Committee (IACUC; protocol no. 3248 and 4026) at Washington State University.

### Construction of 16S rDNA Clone Libraries and Sequence Analysis

Total DNA was isolated from cecal contents using the UltraClean Fecal DNA kit (MoBio Laboratories, Inc, Carlsbad, CA). 16S rRNA sequences were amplified with PCR Super Mix High Fidelity (Invitrogen) as previously described using three sets of primers: 8F and 1492R (Set A), 8F and 1522R (Set B), and 8F and 926R (Set C) [44]. PCR products were pooled and purified using the QiaQuick PCR clean-up kit (Qiagen, Valencia, CA). Purified products were ligated to pCR2.1 (Invitrogen) and transformed into chemically competent *E. coli* TOP10F^-^. Clones were screened for α-complementation of β-galactosidase by using 5-bromo-4-chloro-3-indolyl-β-D-galactopyranoside and isopropyl-β-D-thiogalactopyranoside [45]. Sequencing of constructed libraries was performed at Functional Biosciences, Inc. (Madison, WI) using M13F(−20) and M13R(−27) primers. The resulting sequences were processed and aligned using the Ribosomal Database Project (RDP) pipeline tool (http://rdp.cme.msu.edu) [46]. Aligned sequences were taxonomically classified using the RDP Classifier [47]. Sequences were assigned to operational taxonomic units (OTUs) at 1% sequence dissimilarity using DOTUR [48] on the RapidOTU server (http://genome.jouy.inra.fr/rapidotu). DOTUR was also used to generate the Shannon-Weaver (H’) and Simpson (D) diversity indices for the eight libraries. Evenness (E) was calculated as described previously [49]. Libraries were compared using RDP Library Compare [46].

### Detection of Anti-*C. jejuni* Antibodies in Chick Sera

Plastic 96-well plates were coated with 100 µl of 2 µg/ml *C. jejuni* F38011 whole-cell lysate or *Clostridium perfringens* alpha-toxin (Sigma) diluted in PBS. After incubating plates overnight at 4°C, the wells were washed twice with PBST wash buffer (PBS, 0.05% Tween 20) and blocked with 150 µl of PBS, 0.05% Tween 20, and 0.25% gelatin (PBST-G) at 25°C for 2 h. The plates were washed three times. Chick sera were diluted 1∶200 in PBST-G and 100 µl of each serum sample was added in triplicate. After incubation for 2 h at 25°C, the wells were washed three times and 100 µl of anti-chicken IgG antibody horseradish peroxidase conjugate diluted 1∶5000 in PBST-G was added and incubated for 2 h at 25°C. Wells were washed three times with PBS and 50 µl of tetramethybenzidine substrate (Pierce-Endogen) was added to the wells. The reaction was stopped with 0.18 N H_2_SO_4_ after 10 min of development. Absorbances at 490 nm (A_490_) within wells were determined using an ELx808 Ultra Microplate Reader (BioTek Instruments, Inc., Winooski, VT).

### Detection of Lactic Acid Production

*Lactobacillus* strains were inoculated into ten ml of MRS broth at an O.D._540_ of 0.05 and incubated statically at 37°C in a microaerobic incubator for 48 h. Supernatants were collected from one ml of culture by centrifugation followed by passage through a 0.22 µm filter to remove bacteria. The concentrations of D- and L-lactate in the cell-free supernatants were measured using stereo-specific D- and L-lactate assay kits (Eton Bioscience, San Diego, CA). The measurements were performed in triplicate for reproducibility. Assays were performed with pure solutions of D- and L-lactate to ensure that the kits were stereo-specific.

### Survival of *C. jejuni* Treated with Lactic or Hydrochloric Acid

Survival assays were performed using *C. jejuni* cultures inoculated at an O.D._540_ of 0.2, and incubated at 37°C in a microaerobic incubator with shaking. The MH media used were either untreated, or supplemented with lactic acid at concentrations of 10 mM (pH = 5.12), 25 mM (pH = 4.32), or 100 mM (pH = 3.46). Additional batches of MH media were treated with HCl to pH of 5.12, 4.32, and 3.46. Experiments were performed in triplicate (n = 3), with samples taken at 1, 2, 4, and 8 h post-inoculation. The numbers of viable *C. jejuni* were determined by serial dilution and plating on MHB agar.

### Sample Preparation for FT-IR and Raman Spectroscopy

Fourier transform infrared (FT-IR) and Raman spectroscopies are vibrational spectroscopy techniques that can be used to directly examine the chemical composition of bacterial samples [26]. We chose to use these complementary techniques to observe biochemical changes in bacteria cells, as they are rapid, do not require extensive sample preparation/modification, and yield information on a wide range of chemical structures. Samples for spectroscopic analysis were inoculated into the lactic acid and HCl-treated media described above. Five ml tubes of broth were inoculated with *C. jejuni* or a mixture of all four *Lactobacillus* strains and incubated for 1 h with shaking at 37°C in a microaerobic incubator. For Raman spectroscopic analysis, samples were pelleted by centrifugation, washed once with PBS, and resuspended to an O.D._540_ of 1.0 in PBS. FT-IR and Raman spectroscopy analysis was performed as described previously [50].

### *C. jejuni* and *L. crispatus in vitro* Co-culture Model

We chose to use ultra-pasteurized milk (1% fat) as the medium for co-culture. The milk used in this study was purchased from local grocery stores (n = 3), and combined in equal amounts. Three sterile 125 ml flasks containing 50 ml of pasteurized milk were inoculated; the first with *C. jejuni*, the second with *L. crispatus*, and the third with both *C. jejuni* and *L. crispatus*. The samples were incubated with shaking in a microaerobic environment at 37°C. Samples were taken at 0, 1, 2, 3, 4, 5, 6, 7, 8, 10, 12, 16, and 24 h from all flasks. For monoculture samples, ten-fold serial-dilutions of *C. jejuni* and *L. crispatus* inoculated samples were spread onto the surface of MHB and MRS for viable bacteria enumeration, respectively. For co-culture samples, ten-fold serial-dilutions of the samples were spread onto the surface of Campy-Cefex agar and Rogosa SL agar for viable bacteria enumeration. Samples of the milk cultures were preserved at −80°C, and subsequently used for spectroscopic analyses. The entire experiment was repeated three times. On another day, the previously frozen samples at −80°C were slowly thawed on ice. A 20 µl portion from each sample was then placed onto an aluminum oxide membrane filter, and sample drying was performed under laminar flow at 22°C for 40 min. FT-IR spectra of each sample were collected as described above.

### Spectral Processing and Chemometric Analyses

FT-IR and Raman spectral processing was performed as described previously [50]. A partial least squares regression (PLSR) model was established based upon processed spectra and employed for quantitative analysis in Matlab. A total of 18 spectra of each sample were used to establish the calibration model. To perform model validation, 70% of the total spectra were randomly selected for the training set and the remaining 30% were used as the test set [26]. This selection and test process was repeated three times and the average model values were calculated. The suitability of the developed models for predicting live *C. jejuni* and *L. crispatus* numbers in monoculture and/or co-culture was assessed by regression coefficient (R), latent variables, the root mean square error of estimation (RMSEE), and the root mean square error of cross validation (RMSECV). The overall suitability of the models in predicting the live *C. jejuni* numbers in biofilm was evaluated from the residual prediction deviation (RPD) values [50]. We employed PCA to extract major principal components to investigate the PLSR model for determination of bacterial metabolic activity in pasteurized milk [51]. PCA is a vector space transformation technique for reducing a data set into its predominant features, segregating samples within a data set into discrete clusters [52], [53]. PCA determines the major factors that affect the differences observed in the spectral features among samples and then uses this information to construct a two- or three-dimensional chemometric model to segregate samples on the basis of selected variances.

### *C. jejuni* Growth Curve with Sub-lethal Concentrations of Lactic Acid

An overnight MH broth culture of the *C. jejuni* F38011 strain was used to inoculate 100 ml of MH broth to 0.05 O.D._540_. The culture was incubated with shaking for 3 h to ensure that the *C. jejuni* were in mid-logarithmic growth phase. Five ml aliquots of the culture were split into separate tubes, and supplemented with 0, 5, 10, or 20 mM lactic acid. The number of viable CFU/ml were determined at 0, 1, 2, 4, and 8 h by plating serial dilutions on MHB agar. The assay was performed in triplicate for each treatment.

## Supporting Information

Figure S1 **Inoculation with** ***Lactobacillus*** **does not stimulate production of anti-** ***C. jejuni*** **serum antibodies.** Sera were collected from euthanized broiler chickens 21 days post-hatch and screened for reactivity to *C. jejuni* whole cell lysates. The bars indicate antibody reactivity, black peaks represent *Lactobacillus* colonization, and gray peaks represent *C. jejuni* colonization.
(TIF)

Figure S2 **Inoculation with** ***Lactobacillus*** **does not stimulate production of anti-alpha toxin serum antibodies.** Sera were collected from euthanized broiler chickens 21 days post-hatch and screened for reactivity to *Clostridium perfrinigens* alpha toxin. The bars indicate antibody reactivity, black peaks represent *Lactobacillus* colonization, and gray peaks represent *C. jejuni* colonization.
(TIF)

Figure S3 **Raman spectra segregation models can distinguish between** ***C. jejuni*** **treated with different concentrations of lactic acid.** Raman spectroscopic based cluster analysis and dendrogram models were employed to segregate *C. jejuni* samples according to treatment with selected concentrations of lactic acids. Hierarchical cluster analysis (HCA) models (A and C) and principal component analysis (PCA) models (B and D) using Raman spectra were used to segregate *C. jejuni* untreated (sample 1–10 in A and C and category A in B and D) and treated with 25 mmol/L (sample 11–20 in A and C and category B in B and D) and 100 mmol/L (sample 21–30 in A and C and category C in B and D) lactic acid. Raman spectra were used from three independent experiments (n = 3). Each group was clearly distinguished from each other, forming tight clusters with interclass distances ranging from 9.58 to 36.31, based on *Mahalanobis* distance measurements computed between the centroids of classes. Clusters with interclass distance values higher than 3 are considered to be significantly different from each other.
(TIF)

Figure S4 **Representative** **FT-IR spectra of bacteria in co-culture.** Typical FT-IR spectra at 8 h for *C. jejuni* and *L. crispatus* after inoculation in pasteurized milk in monoculture and in co-culture. Spectra are offset so that features can be observed.
(TIF)

Figure S5 **A partial least squares regression model to validate the use of FT-IR spectra to predict bacterial viability.** Quantification of each bacterial species was performed using a partial least squares regression (PLSR) model. Each model was constructed using 70% of the values as the training set and the remaining 30% for the validation set. Another batch of spectral data was used as the prediction set. Eight different models were constructed using different combinations of spectra in the training and validation sets, and these results were combined to obtain the average prediction statistics. Only data collected from the first 720 min of the experiments were used, as similar CFU for both *C. jejuni* and *L. crispatus* in monoculture and co-culture were observed during this period. Two representative PLSR models predicting *C. jejuni* numbers from pure cultures (Panel A) and co-cultures with *L. crispatus* (Panel B).
(TIF)

Figure S6 **Sub-lethal concentrations of lactic acid inhibit** ***C. jejuni*** **metabolism.** Mid-log phase cultures of *C. jejuni* were treated with 0, 5, 10, and 20 mM lactic acid, and viable CFU/mL were enumerated at 0, 1, 2, 4, and 8 h. The data points represent the average of three experimental replicates and error bars represent one standard deviation from the mean.
(TIF)

Table S1 **Bacterial strains used in this study.** ^a^Japan Collection of Microorganisms. ^b^American Type Culture Collection. ^c^Centre National de Recherche Zootechnique.
(DOC)

Table S2 **Inhibition of** ***C. jejuni*** **by lactobacilli^a^.** ^a^Expressed as Ratio of Zone of Inhibition (mm)/Zone of Growth (mm). ^b^± represent one standard deviation from the mean of triplicate assays.
(DOCX)

Table S3 **Features of specimens selected for cecal microbiome analysis.** ^a^Counts shown as CFU/gm of cecal or ileal contents ^b^Positve control for *C. jejuni* colonization, receiving only *C. jeuni* ^c^ND – not detected, limit of detection is 1×10^3 ^CFU/gm.
(DOCX)

Table S4 **Taxonomic distribution of rRNA gene clones obtained from 16s PCR analysis of chicken ceca.** ^a^Percents indicate the ration of clones within each library. ^b^OTU, operational taxonomic units, where sequences with ≥99% nucleotide identity are considered an OTU.
(DOCX)

Table S5 **Comparison of band assignments of significant (** ***P*** **<0.05) spectral variations of** ***C. jejuni*** **treated with lactic acid and hydrochloric acid.** ^a^sym = symmetric; str = stretching.
(DOCX)

Table S6 **Comparison of the PLS regression results of the FT-IR spectra for determining the number of viable bacteria in pasteurized milk.** ^a^The wavenumbers from 3300 to 2700 cm^−1^ and 1800 to 700 cm^−1^ were used for model analyses. ^b^RMSE cal: root mean square error for calibration. ^c^RMSE val: mean square error for cross validation. ^d^RPD: residual prediction deviation.
(DOC)
