# Supplementary figures and images for: Production of Organic Acids by Probiotic Lactobacilli Can Be Used to Reduce Pathogen Load in Poultry
Source: PLoS One. 2012 Sep 4;7(9):e43928. doi: 10.1371/journal.pone.0043928 (PMC3433458; doi:10.1371/journal.pone.0043928)

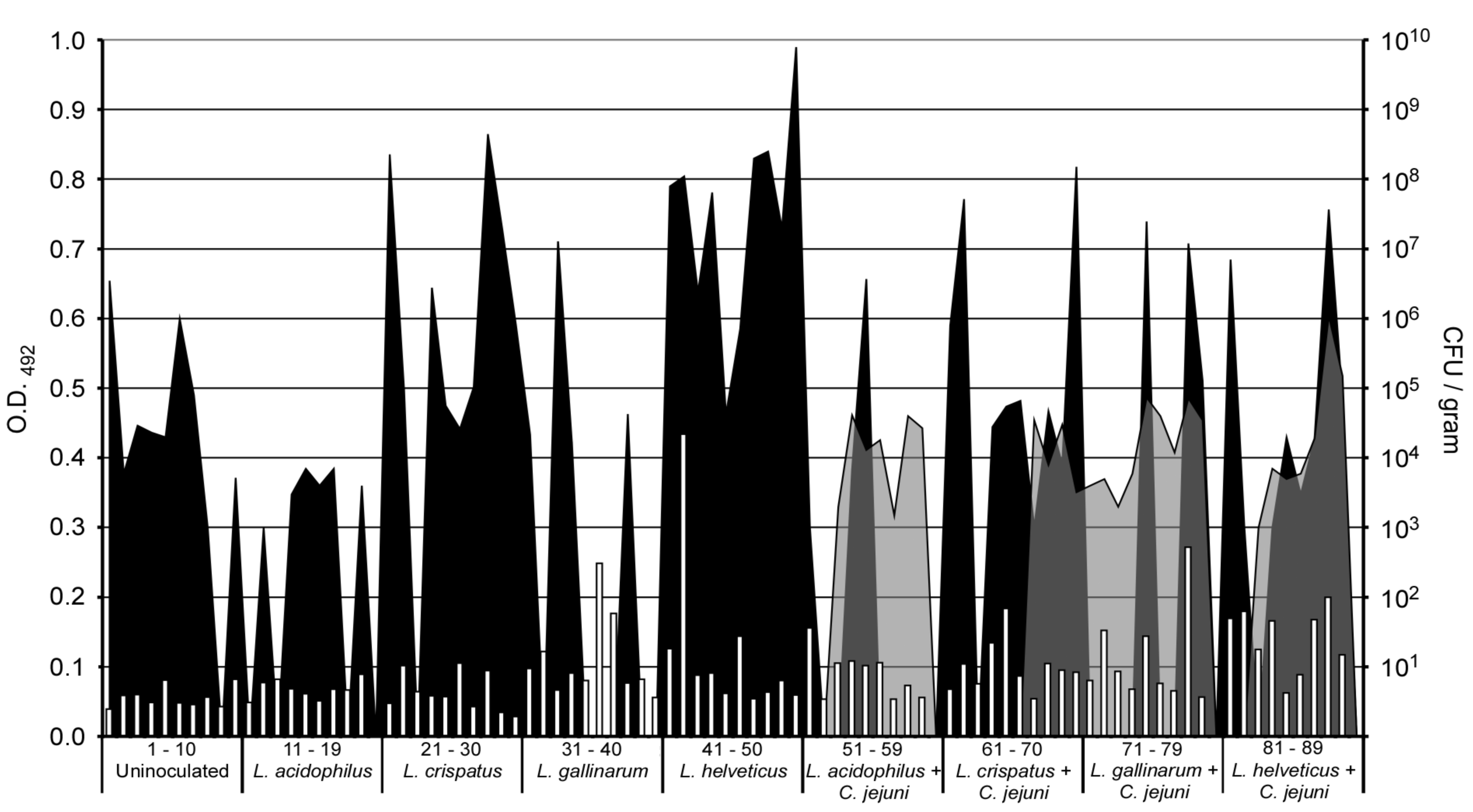

Supplement: Figure S1 — Inoculation with Lactobacillus does not stimulate production of anti- C. jejuni serum antibodies. Sera were collected from euthanized broiler chickens 21 days post-hatch and screened for reactivity to C. jejuni whole cell lysates. The bars indicate antibody reactivity, black peaks represent Lactobacillus colonization, and gray peaks represent C. jejuni colonization. (TIF) [file pone.0043928.s001.tif]

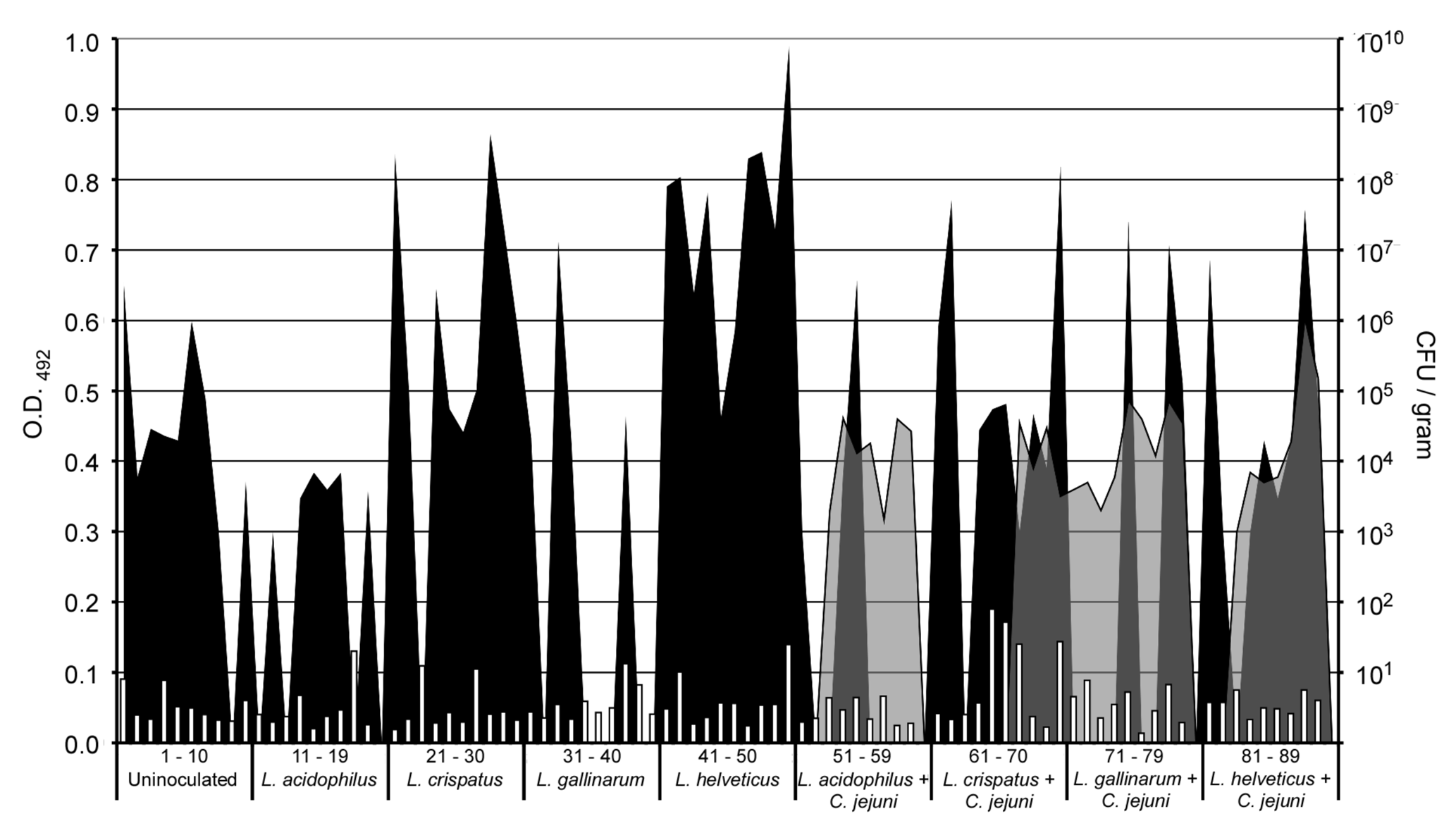

Supplement: Figure S2 — Inoculation with Lactobacillus does not stimulate production of anti-alpha toxin serum antibodies. Sera were collected from euthanized broiler chickens 21 days post-hatch and screened for reactivity to Clostridium perfrinigens alpha toxin. The bars indicate antibody reactivity, black peaks represent Lactobacillus colonization, and gray peaks represent C. jejuni colonization. (TIF) [file pone.0043928.s002.tif]

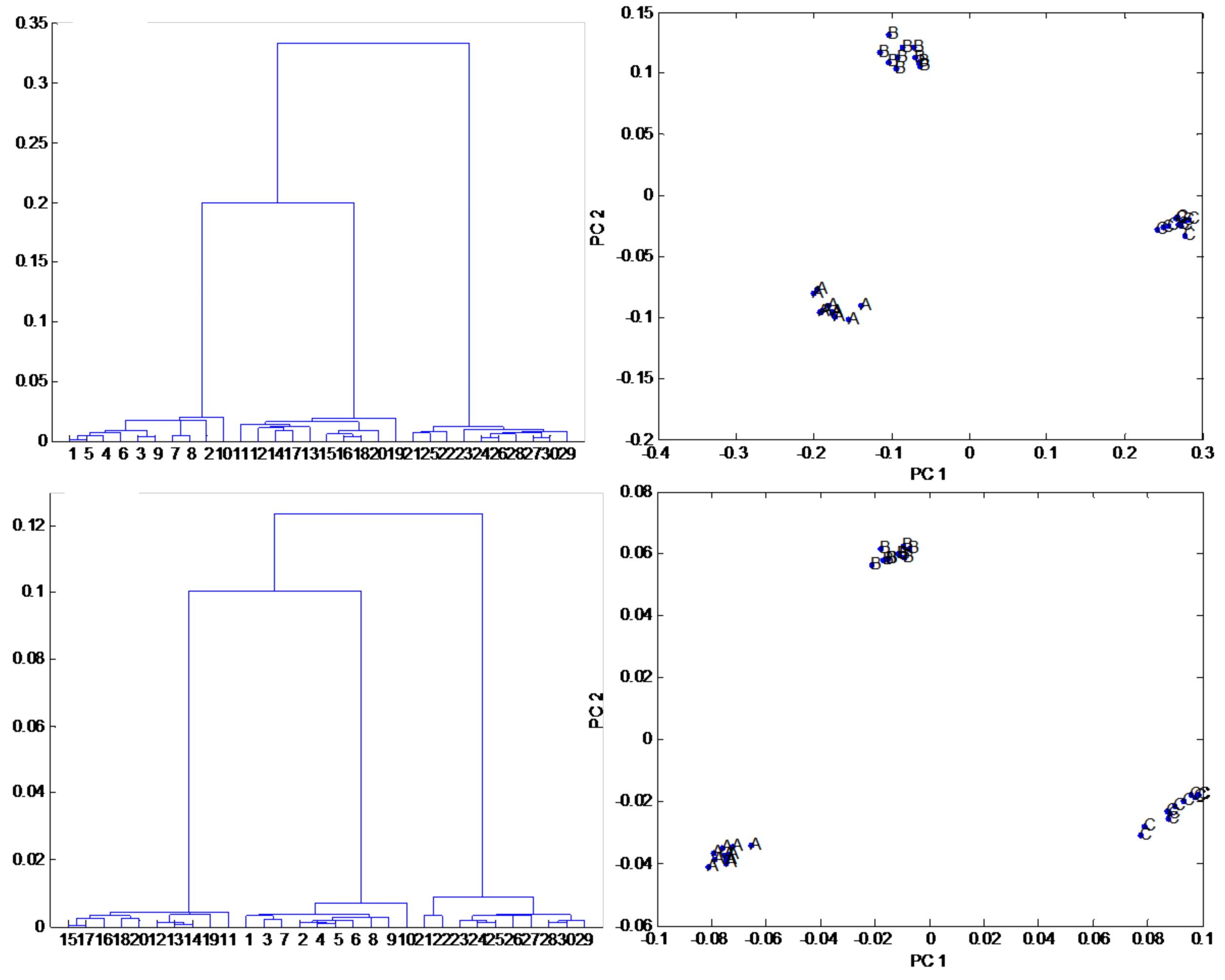

Supplement: Figure S3 — Raman spectra segregation models can distinguish between C. jejuni treated with different concentrations of lactic acid. Raman spectroscopic based cluster analysis and dendrogram models were employed to segregate C. jejuni samples according to treatment with selected concentrations of lactic acids. Hierarchical cluster analysis (HCA) models (A and C) and principal component analysis (PCA) models (B and D) using Raman spectra were used to segregate C. jejuni untreated (sample 1–10 in A and C and category A in B and D) and treated with 25 mmol/L (sample 11–20 in A and C and category B in B and D) and 100 mmol/L (sample 21–30 in A and C and category C in B and D) lactic acid. Raman spectra were used from three independent experiments (n = 3). Each group was clearly distinguished from each other, forming tight clusters with interclass distances ranging from 9.58 to 36.31, based on Mahalanobis distance measurements computed between the centroids of classes. Clusters with interclass distance values higher than 3 are considered to be significantly different from each other. (TIF) [file pone.0043928.s003.tif]

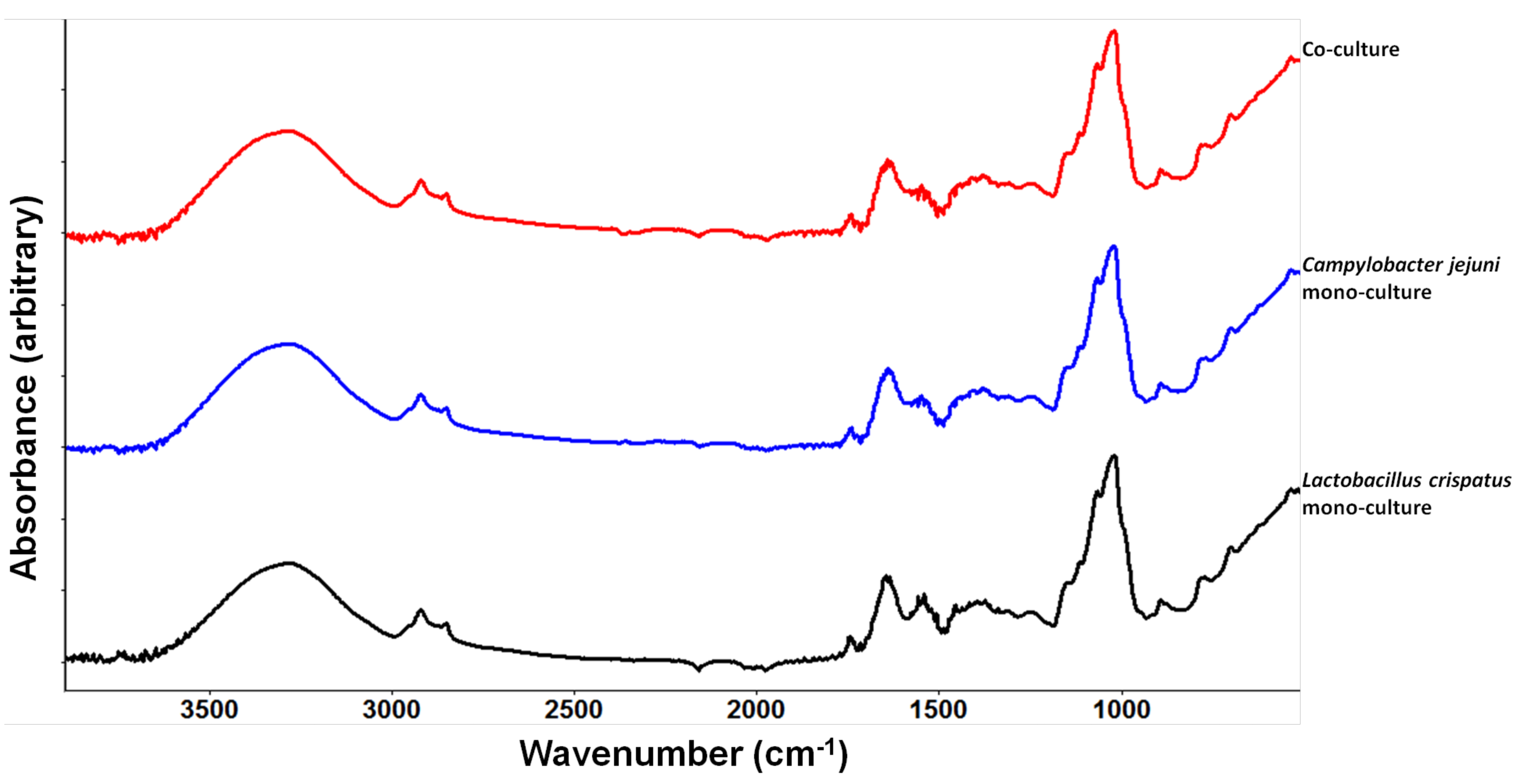

Supplement: Figure S4 — Representative FT-IR spectra of bacteria in co-culture. Typical FT-IR spectra at 8 h for C. jejuni and L. crispatus after inoculation in pasteurized milk in monoculture and in co-culture. Spectra are offset so that features can be observed. (TIF) [file pone.0043928.s004.tif]

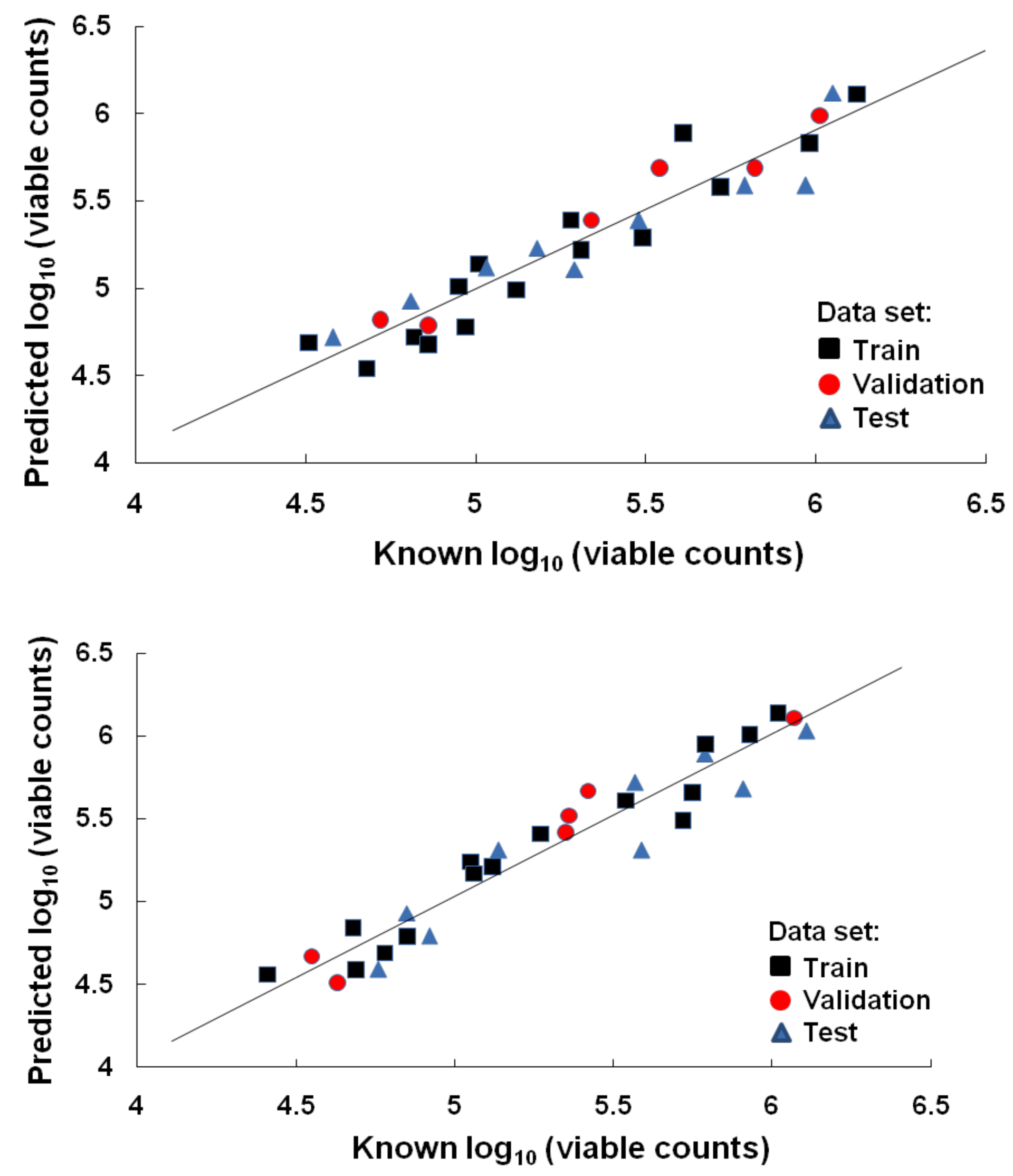

Supplement: Figure S5 — A partial least squares regression model to validate the use of FT-IR spectra to predict bacterial viability. Quantification of each bacterial species was performed using a partial least squares regression (PLSR) model. Each model was constructed using 70% of the values as the training set and the remaining 30% for the validation set. Another batch of spectral data was used as the prediction set. Eight different models were constructed using different combinations of spectra in the training and validation sets, and these results were combined to obtain the average prediction statistics. Only data collected from the first 720 min of the experiments were used, as similar CFU for both C. jejuni and L. crispatus in monoculture and co-culture were observed during this period. Two representative PLSR models predicting C. jejuni numbers from pure cultures (Panel A) and co-cultures with L. crispatus (Panel B). (TIF) [file pone.0043928.s005.tif]

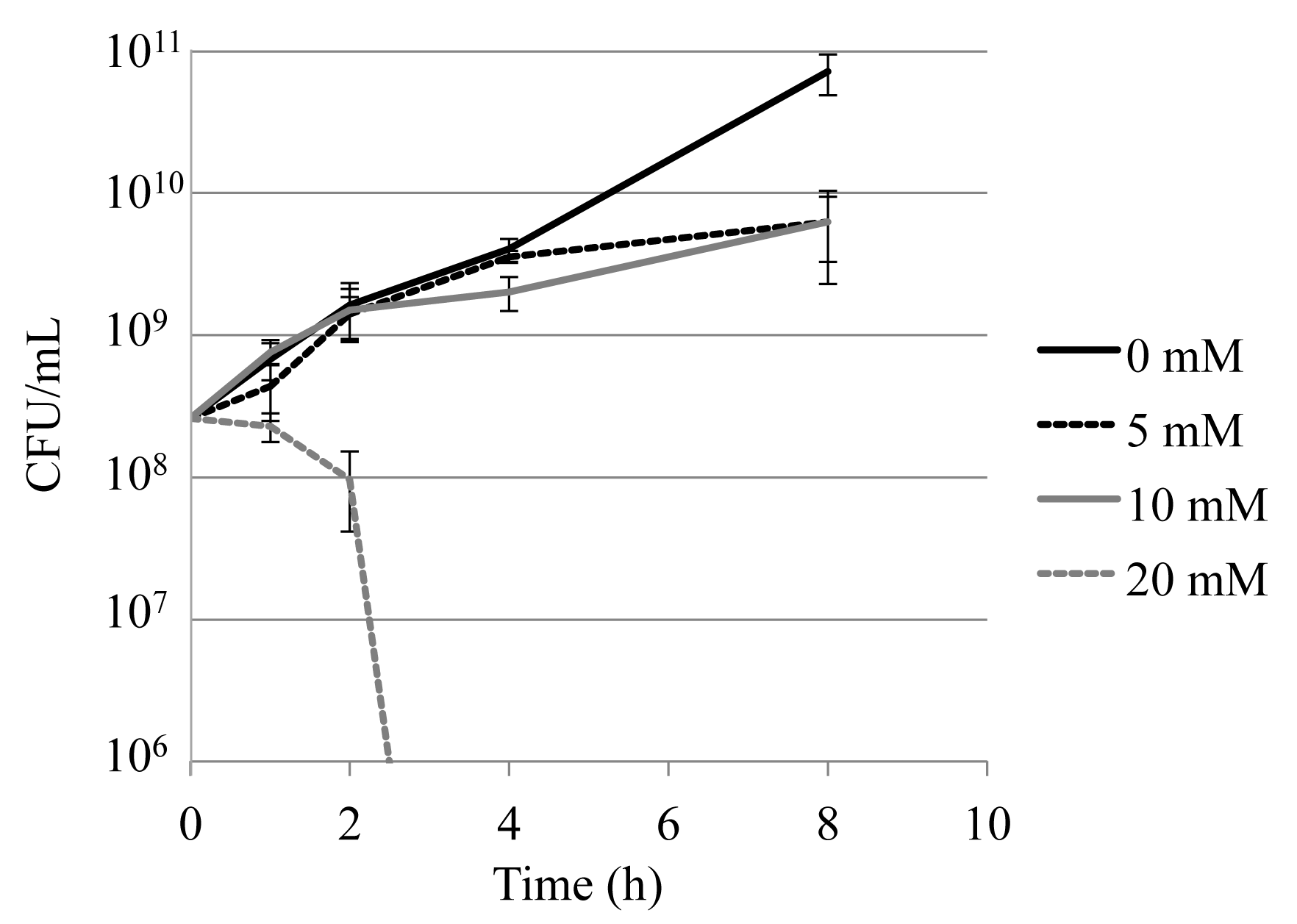

Supplement: Figure S6 — Sub-lethal concentrations of lactic acid inhibit C. jejuni metabolism. Mid-log phase cultures of C. jejuni were treated with 0, 5, 10, and 20 mM lactic acid, and viable CFU/mL were enumerated at 0, 1, 2, 4, and 8 h. The data points represent the average of three experimental replicates and error bars represent one standard deviation from the mean. (TIF) [file pone.0043928.s006.tif]
