# Supplementary material for: Production of Organic Acids by Probiotic Lactobacilli Can Be Used to Reduce Pathogen Load in Poultry
Source: PLoS One. 2012 Sep 4;7(9):e43928. doi: 10.1371/journal.pone.0043928 (PMC3433458; doi:10.1371/journal.pone.0043928)
Supplement: Table S1 — Bacterial strains used in this study. aJapan Collection of Microorganisms. bAmerican Type Culture Collection. cCentre National de Recherche Zootechnique. (DOC) [file pone.0043928.s007.doc]

Table S1. Bacterial strains used in this study.

| Strain | Relevant Characteristics | Source or Reference |
| --- | --- | --- |
| *C. jejuni* |  |  |
| F38011 | Human clinical isolate | [1] |
| 81-176 | Human clinical isolate | [2] |
| 81116 | Human clinical isolate | [3] |
| RM1221 | Poultry isolate | [4] |
| S2B | Poultry isolate | [5] |
| Turkey | Poultry isolate | [5] |
| *Caulobacter crescentus* JS4022 | Laboratory strain | [6] |
| *E. coli* TOP10F^-^ | Cloning host | Invitrogen |
| *L. acidophilus* NCFM | Human isolate | [7] |
| *L. crispatus* JCM 5810 | Chicken isolate | JCM^a^ |
| *L. gallinarum* ATCC 33199 | Chicken isolate, Neotype Strain | ATCC^b^ |
| *L. helveticus* CNRZ32 | Dairy starter strain | CNRZ^c^ |

^a^ Japan Collection of Microorganisms.

^b^ American Type Culture Collection.

^c^ Centre National de Recherche Zootechnique.

**References**

1. Konkel ME, Mead DJ, Cieplak W Jr. (1993) Kinetic and antigenic characterization of altered protein synthesis by *Campylobacter jejuni* during cultivation with human epithelial cells. J Infect Dis 168: 948-954.
2. Hofreuter D, Tsai J, Watson RO, Novik V, Altman B, et al. (2006) Unique features of a highly pathogenic *Campylobacter jejuni* strain. Infect Immun 74: 4694-4707.
3. Pearson BM, Gaskin DJ, Segers RP, Wells JM, Nuijten PJ, et al. (2007) The complete genome sequence of *Campylobacter jejuni* strain 81116 (NCTC11828). J Bacteriol 189: 8402-8403.
4. Fouts DE, Mongodin EF, Mandrell RE, Miller WG, Rasko DA, et al. (2005) Major structural differences and novel potential virulence mechanisms from the genomes of multiple *Campylobacter* species. PLoS Biol 3: e15.
5. Malik-Kale P, Raphael BH, Parker CT, Joens LA, Klena JD, et al. (2007) Characterization of genetically matched isolates of *Campylobacter jejuni* reveals that mutations in genes involved in flagellar biosynthesis alter the organism's virulence potential. Appl Environ Microbiol 73: 3123-3136.
6. Nomellini JF, Duncan G, Dorocicz IR, Smit J (2007) S-layer-mediated display of the immunoglobulin G-binding domain of streptococcal protein G on the surface of *Caulobacter crescentus*: development of an immunoactive reagent. Appl Environ Microbiol 73: 3245-3253.
7. Altermann E, Russell WM, Azcarate-Peril MA, Barrangou R, Buck BL, et al. (2005) Complete genome sequence of the probiotic lactic acid bacterium *Lactobacillus acidophilus* NCFM. Proc Natl Acad Sci U S A 102: 3906-3912.
