# Supplementary material for: Production of Organic Acids by Probiotic Lactobacilli Can Be Used to Reduce Pathogen Load in Poultry
Source: PLoS One. 2012 Sep 4;7(9):e43928. doi: 10.1371/journal.pone.0043928 (PMC3433458; doi:10.1371/journal.pone.0043928)
Supplement: Table S2 — Inhibition of C. jejuni by lactobacillia. aExpressed as Ratio of Zone of Inhibition (mm)/Zone of Growth (mm). b± represent one standard deviation from the mean of triplicate assays. (DOCX) [file pone.0043928.s008.docx]

**Table S2. Inhibition of *C. jejuni* by lactobacilli^a^.**

|  | *C. jejuni* Strains | | | | | |
| --- | --- | --- | --- | --- | --- | --- |
| *Lactobacillus* Strains | F38011 | 81-176 | 81116 | RM1221 | S2B | Turkey |
| *L. acidophilus* | 2.6 ± 0.3^b^ | 1.6 ± 0.1 | 1.5 ± 0.1 | 1.7 ± 0.1 | 1.5 ± 0.1 | 1.9 ± 0.1 |
| *L. crispatus* | 4.1 ± 0.8 | 1.8 ± 0.1 | 1.4 ± 0.2 | 1.9 ± 0.2 | 1.4 ± 0.2 | 1.9 ± 0.3 |
| *L. gallinarum* | 2.4 ± 0.4 | 1.2 ± 0.1 | 1.1 ± 0.1 | 1.2 ± 0.1 | 1.0 ± 0.1 | 1.3 ± 0.2 |
| *L. helveticus* | 1.5 ± 0.1 | 1.2 ± 0.1 | 1.2 ± 0.1 | 1.2 ± 0.1 | 1.2 ± 0.1 | 1.3 ± 0.1 |

^a^ Expressed as Ratio of Zone of Inhibition (mm) / Zone of Growth (mm).

^b^ ± represent one standard deviation from the mean of triplicate assays.
