# Supplementary material for: Production of Organic Acids by Probiotic Lactobacilli Can Be Used to Reduce Pathogen Load in Poultry
Source: PLoS One. 2012 Sep 4;7(9):e43928. doi: 10.1371/journal.pone.0043928 (PMC3433458; doi:10.1371/journal.pone.0043928)
Supplement: Table S3 — Features of specimens selected for cecal microbiome analysis. aCounts shown as CFU/gm of cecal or ileal contents bPositve control for C. jejuni colonization, receiving only C. jeuni cND – not detected, limit of detection is 1×10^3^ CFU/gm. (DOCX) [file pone.0043928.s009.docx]

**Table S3. Features of specimens selected for cecal microbiome analysis.**

|  |  | Bacterial Counts^a^ | | | |
| --- | --- | --- | --- | --- | --- |
| Specimen # | Treatment | *Campylobacter* | | *Lactobacillus* | |
|  |  | Cecum | Illeum | Cecum | Illeum |
| 6 | Uninoculated | ND^c^ | ND | 4.0 × 10^4^ | 4.0 × 10^3^ |
| 21 | *L. crispatus* | ND | ND | 1.9 × 10^9^ | 2.3 × 10^6^ |
| 31 | *L. gallinarum* | ND | ND | 1.5 × 10^7^ | ND |
| 41 | *L. helveticus* | ND | ND | 2.5 × 10^8^ | 3.0 × 10^6^ |
| 68 | *L. crispatus + C. jejuni* | 2.0 × 10^4^ | ND | 4.8 × 10^7^ | 5.2 × 10^7^ |
| 78 | *L. helveticus + C. jejuni* | 2.0 × 10^4^ | ND | 2.2 × 10^6^ | 1.3 x 10^5^ |
| 89 | *L. gallinarum + C. jejuni* | ND | ND | 3.5 × 10^8^ | 9.1 x 10^6^ |
| C^b^ | *C. jejuni* | 2.0 × 10^6^ | ND | ND | ND |

^a^ Counts shown as CFU/gm of cecal or ileal contents

^b^ Positve control for *C. jejuni* colonization, receiving only *C. jeuni*

^c^ ND – not detected, limit of detection is 1 x 10^3^ CFU/gm
