# Supplementary material for: Production of Organic Acids by Probiotic Lactobacilli Can Be Used to Reduce Pathogen Load in Poultry
Source: PLoS One. 2012 Sep 4;7(9):e43928. doi: 10.1371/journal.pone.0043928 (PMC3433458; doi:10.1371/journal.pone.0043928)
Supplement: Table S4 — Taxonomic distribution of rRNA gene clones obtained from 16s PCR analysis of chicken ceca. aPercents indicate the ration of clones within each library. bOTU, operational taxonomic units, where sequences with ≥99% nucleotide identity are considered an OTU. (DOCX) [file pone.0043928.s010.docx]

**Table S4. Taxonomic distribution of rRNA gene clones obtained from 16s PCR analysis of chicken ceca.**
