# Supplementary material for: Production of Organic Acids by Probiotic Lactobacilli Can Be Used to Reduce Pathogen Load in Poultry
Source: PLoS One. 2012 Sep 4;7(9):e43928. doi: 10.1371/journal.pone.0043928 (PMC3433458; doi:10.1371/journal.pone.0043928)
Supplement: Table S5 — Comparison of band assignments of significant ( P <0.05) spectral variations of C. jejuni treated with lactic acid and hydrochloric acid. asym = symmetric; str = stretching. (DOCX) [file pone.0043928.s011.docx]

**Table S5. Comparison of band assignments of significant (*P* < 0.05) spectral variations of *C. jejuni* treated with lactic acid and hydrochloric acid.**

| *C. jejuni* treated with lactic acid | | | | *C. jejuni* treated with hydrochloric acid | |
| --- | --- | --- | --- | --- | --- |
| FT-IR | | Raman | | FT-IR | |
| Wavenumber (cm^-1^) | Assignment ^a^ | Wavenumber (cm^-1^) | Assignment | Wavenumber (cm^-1^) | Assignment |
| 929 | left-hand helix DNA | 898 | C-O-C of mono/di-saccharides | 985 | OCH_3_ of polysaccharides |
| 985 | OCH_3_ of polysaccharides | 1008 | ring of polysaccharides | 1078 | C-OH str of oligosaccharides |
| 1078 | C-OH str of oligosaccharide | 1241 | phosphodiester of nucleic acids | 1545 | amide II |
| 1396 | sym CH_3_ bend of proteins | 1420 | deoxyriboses | 1635 | *β* sheet structure of amide I |
| 1444 | *δ*(CH_2_) lipids and fatty acids | 1745 | *υ*(C=O) of phospholipids | 1655 | amide I of proteins (*α*-helix) |
| 1465 | CH_2_ scissoring mode of lipids | 1778 | *unassigned* |  |  |
| 1655 | amide I of proteins (*α*-helix) |  |  |  |  |

^a^ sym=symmetric; str=stretching.
