# Supplementary material for: Production of Organic Acids by Probiotic Lactobacilli Can Be Used to Reduce Pathogen Load in Poultry
Source: PLoS One. 2012 Sep 4;7(9):e43928. doi: 10.1371/journal.pone.0043928 (PMC3433458; doi:10.1371/journal.pone.0043928)
Supplement: Table S6 — Comparison of the PLS regression results of the FT-IR spectra for determining the number of viable bacteria in pasteurized milk. aThe wavenumbers from 3300 to 2700 cm−1 and 1800 to 700 cm−1 were used for model analyses. bRMSE cal: root mean square error for calibration. cRMSE val: mean square error for cross validation. dRPD: residual prediction deviation. (DOC) [file pone.0043928.s012.doc]

Supplemental Table 6. Comparison of the PLS regression results of the FT-IR spectra for determining the number of viable bacteria in pasteurized milk, related to Figure 6.

| Spectra ^a^ | Range (CFU/mL) | No. of samples | No. of latent variables | R cal | RMSE cal ^b^ | R val | RMSE val ^c^ |
| --- | --- | --- | --- | --- | --- | --- | --- |
| *C. jejuni* monoculture | 4.45-6.13 | 528 | 5 | 0.95 | ≤0.33 | 0.94 | ≤0.45 |
| *L. crispatus* monoculture | 4.67-6.21 | 528 | 4 | 0.96 | ≤0.28 | 0.95 | ≤0.39 |
| *C. jejuni* in coculture | 4.78-6.16 | 520 | 6 | 0.95 | ≤0.39 | 0.92 | ≤0.48 |
| *L. crispatus* in coculture | 4.52-6.28 | 520 | 5 | 0.95 | ≤0.28 | 0.94 | ≤0.36 |

^a^ The wavenumbers from 3300 to 2700 cm^-1^ and 1800 to 700 cm^-1^ were used for model analyses.

^b^ RMSE cal: root mean square error for calibration.

^c^ RMSE val: mean square error for cross validation.

^d^ RPD: residual prediction deviation.
